# Role of hydrogen in volatile behaviour of defects in SiO_2_-based electronic devices

**DOI:** 10.1098/rspa.2016.0009

**Published:** 2016-06

**Authors:** Yannick Wimmer, Al-Moatasem El-Sayed, Wolfgang Gös, Tibor Grasser, Alexander L. Shluger

**Affiliations:** 1 Institute for Microelectronics, Vienna University of Technology, Gußhausstraße 27–29/E360, 1040 Wien, Austria; 2 Department of Physics and Astronomy and London Centre for Nanotechnology, University College London, Gower Street, London WC1E 6BT, UK

**Keywords:** silica defects, multiscale modelling, NBTI, RTN

## Abstract

Charge capture and emission by point defects in gate oxides of metal–oxide–semiconductor field-effect transistors (MOSFETs) strongly affect reliability and performance of electronic devices. Recent advances in experimental techniques used for probing defect properties have led to new insights into their characteristics. In particular, these experimental data show a repeated dis- and reappearance (the so-called *volatility*) of the defect-related signals. We use multiscale modelling to explain the charge capture and emission as well as defect volatility in amorphous SiO_2_ gate dielectrics. We first briefly discuss the recent experimental results and use a multiphonon charge capture model to describe the charge-trapping behaviour of defects in silicon-based MOSFETs. We then link this model to *ab initio* calculations that investigate the three most promising defect candidates. Statistical distributions of defect characteristics obtained from *ab initio* calculations in amorphous SiO_2_ are compared with the experimentally measured statistical properties of charge traps. This allows us to suggest an atomistic mechanism to explain the experimentally observed volatile behaviour of defects. We conclude that the hydroxyl-E′ centre is a promising candidate to explain all the observed features, including defect volatility.

## 1. Introduction

The performance of metal–oxide–semiconductor field-effect transistors (MOSFETs) is affected by a number of detrimental factors, such as random telegraph noise (RTN) [1,2], 1/f noise [3] and bias temperature instability (BTI) [4–7]. Although these effects have been studied for more than 40 years, the underlying physical mechanisms are still controversial [8,9]. Their impact on overall device performance becomes ever more prominent as MOSFET sizes scale down and reach nanometre dimensions. Although it is widely accepted that these effects are caused by trapping and release of electrons and holes by defects from a semiconductor channel of an MOSFET (typically Si) [1,10,11], the detailed microscopic nature of these defects remains unknown.

In large devices, an ensemble of defects continuously trap and emit charges. Consequently, the individual defect properties are averaged out in experimental data and unambiguous identification of the underlying mechanisms is difficult. With the advances in MOSFET technology during the last several years, device dimensions have been continuously downsized and have now reached a point where device degradation is dominated by the occurrence of single charging or discharging events [11–16]. This has aggravated the impact of individual defects on the devices’ behaviour as well as leading to performance variations between devices of the same kind (which is often referred to as time-dependent variability). On the other hand, these advances also led to the development of new experimental methods for probing individual defect properties. Using small-area devices, which typically contain very few defects, one can now study the electrical response to charge capture and emission by single defects. In such measurements, one can clearly and unambiguously identify and characterize the individual defects responsible for the macroscopically measurable behaviour.

The capture and emission of carriers at individual defect sites generates discrete changes in the conductance of electronic devices, referred to as a random telegraph noise/signal (RTN). Analysing RTN can, therefore, be used to provide information on charge-trapping defects in the oxide. Unfortunately, this is feasible only for defects with similar capture and emission times [11]. Time-dependent defect spectroscopy (TDDS), on the other hand, does not have this limitation and allows one to study charge-trapping dynamics by individual defects in a systematic manner. Recent TDDS experiments on the defects responsible for charge capture in oxides [17,18] have revealed that defects often exhibit a metastability in both the neutral and positively charged states. Some defects have been found to behave like switching oxide traps [19], whereas others exhibit voltage-independent emission time constants. Moreover, some defects were found to be *volatile*, becoming electrically inactive for random amounts of time, a curious feature previously observed for RTN [20,21].

In this paper, we focus on explaining the volatility in the behaviour of oxide defects observed in electrical RTN and TDDS measurements using a computational method combining a phenomenological non-radiative multiphonon (NMP) model of electron transfer with *ab initio* calculations of defect properties. We first briefly discuss the recent TDDS results and use a multiphonon charge capture model to describe the trapping behaviour of defects in silicon-based MOSFETs. We then briefly review the results of density functional theory (DFT) calculations of defects which are likely candidates for charge capture in the SiO_2_ layer of MOSFETs and discuss possible mechanisms of the temperature-activated dynamics responsible for the observed volatility. Our results demonstrate a complex interplay of electron capture/emission and thermally activated hydrogen motion in oxide films.

## 2. Defect characterization

### (a) Time-dependent defect spectroscopy

Oxide defects can be charged and discharged when a charge transition level of a defect is moved across the Fermi-level of the system. In electronic devices, this happens when a certain stress–voltage (*bias*) is applied across the oxide of a device. Charge capture and emission time constants of oxide defects are distributed over many orders of magnitude, from nano- to several kilo-seconds (and presumably even more, because our measurement window is limited to these time scales) [1,11,17]. In large devices, the measured electrical signals correspond to collective response of a large number of defects [1,22,23], making it difficult to study charge capture and emission by single defects. Downscaling of devices reduces the number of defects per device dramatically. This makes probing of individual defects (for example using the TDDS method) much easier.

**Figure 1.**
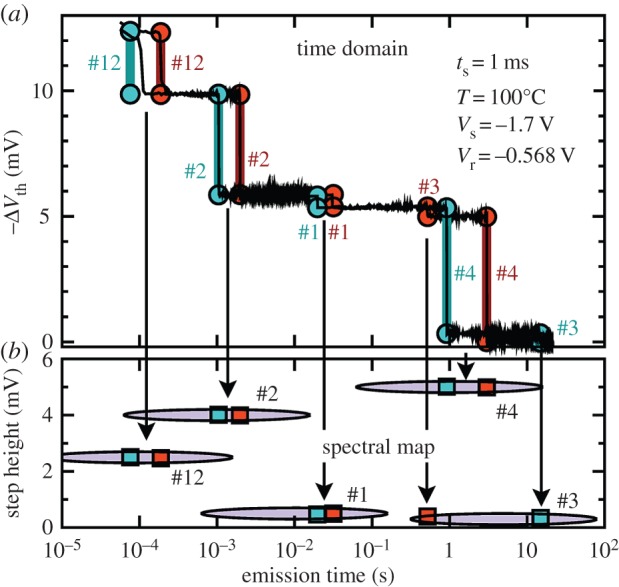
Two typical Δ*V*_th_ recovery traces of a small-area pMOSFET from a TDDS experiment. The measured data are given by the noisy black lines (*a*). The thick blue and red lines together with the symbols mark the emission times and step heights, unambiguous fingerprints of each defect which constitute the spectral map (*b*).

TDDS allows one to analyse defects with widely different capture and emission behaviour over an extremely wide range of time constants [17,24]. These experiments require the use of small-area devices (typically 100×100 nm and smaller) that usually contain less than 10 defects in the gate oxide. Under these circumstances, one can clearly observe the charging dynamics of individual defects. TDDS experiments involve stressing the device for particular periods of time by application of a suitable stress voltage. As a result, some of the defects in the device trap charge (capturing either an electron or a hole) leading to a shift in threshold voltage, Δ*V*_th_. When the stress is removed, defects can emit their charge, with each such event leading to a discrete shift in the threshold voltage of a device. A recovery trace of the threshold voltage is therefore recorded after the stress is removed with each discrete shift representing the emission of a charge (see red and blue bars in figure 1) [11,17,24–26]. This discrete shift in the threshold voltage is referred to as a step height and is caused by charged or discharged defects, altering the electrostatics of the device. The step height does not depend on the microscopic structure of the defect but rather on the position of the charge in the oxide and its interaction with the potential inside the channel [10,27]. However, the time at which the defect emits the charge *is* determined by the atomic structure of the defect. Figure 1 shows two typical TDDS traces. The step heights are clearly observed in the top panel and are marked by numbers. Figure 1 shows two different runs on the same device, where the discrete step heights occur at different emission times (in the second trace, for example, the emission of #4 even occurs before #3). Provided that the number of defects is small, they can be unambiguously identified by their step height and emission time.

**Figure 2.**
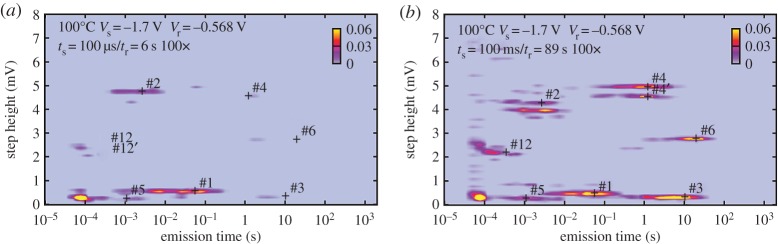
Two TDDS spectral maps at two stress times, (*a*) *t*_s_=100 μs and (*b*) *t*_s_=10 ms. With increasing stress time, the number of defects in the map increases. The width of each cluster is given by the exponential distribution of *τ*_e_ (considered on a log scale) and the extracted defects/clusters are marked by ‘plus’ symbols.

Charge emission during recovery is a stochastic event [14,15,28–30]. Therefore, for reliable characterization, a relatively large number of stress/recovery experiments have to be performed on the same device. The statistical properties of the discrete steps in the recovery traces can then be analysed by collecting the step heights and emission times, *τ*_e_, of each emission event (figure 1). The accumulated pairs are then binned into a two-dimensional histogram (figure 1, bottom). Performing this many times results in the spectral maps depicted for typical cases in figure 2. The bright clusters are indicative of a single defect, and one can see a number of different defects with very different emission times within one device. The two example TDDS spectral maps at the two stress times, *t*_s_=100 μs (figure 2*a*) and *t*_s_=10 ms (figure 2*b*), demonstrate that for increasing stress time, the number of defects in the map increases, meaning that more defects become populated. TDDS experiments on the same device using different stress voltages, stress times and temperatures provide a wealth of information regarding the dynamics of electron/hole capture and emission by individual defects, which can be used for identifying the defects involved.

Figure 1 shows the case for negative-BTI (NBTI) in a pMOS device. Similar traces for nMOS devices and the positive-BTI (PBTI) effect can be seen in references [31–33]. However, in this work, we focus on the NBTI effect in pMOS devices because it is far more pronounced than its PBTI counterpart in nMOS devices [7,34]. Nevertheless, it should be pointed out that the model developed in the following section is applicable to both NBTI and PBTI.

### (b) Theoretical charge capture models

Many different models have been previously proposed in the literature to explain the results of charge capture and emission measurements. The early models relied on elastic carrier tunnelling between the substrate and oxide defects [35–39]. However, these models show negligible temperature and bias dependence, in contrast to experimental observations. Other models are based on the well-known Shockley–Read–Hall (SRH) model [40] modified to account for the tunnelling effect [41] and the thermal activation seen in RTN data [10,42]. For example, the model employed in the pioneering work by Kirton & Uren [10] is one such model that is still widely used. It recognizes that the SRH model is unable to explain the experimental data because it ignores the lattice deformation around the defect site when the charge state is changed and that NMP processes should be included. In order to account for these processes, Kirton and Uren introduced a Boltzmann factor into the SRH rates to account for structural relaxation [10]. However, the very strong bias dependence of this term was neglected in this early work.

The approach by Kirton and Uren has been further developed in the framework of NMP transition rate theory widely used in electrochemistry and to describe electron transfer processes in solids and solutions [43,44]. To explain the thermal activation of NBTI, transition barriers were phenomenologically introduced to reproduce the observed temperature dependence [10,42] justified by the importance of NMP transitions. However, ensuing relations were not rigorously derived from a microscopic theory [45–55].

Here, we summarize our recent efforts to explain the results obtained from TDDS measurements using an NMP model (whose details are published elsewhere [11,17,56]). This model provides us a more rigorous framework for the description of the charge-transfer process between the substrate and the oxide defects, and was introduced in the context of RTN and NBTI in the work of Grasser *et al.* [11,26]. As semiconductor devices are typically operated at room temperature and above, a semi-classical version of the theory is used. It uses the diabatic approximation to describe the electron transfer between the substrate and defect states within the harmonic approximation. In this approximation, the description of the model is similar to the conventional language of Marcus parabolas [57]. In oxide traps, the relative vertical position of the parabolas changes with bias, thereby naturally introducing the required strong bias dependence into the model, in addition to some other features.

**Figure 3.**
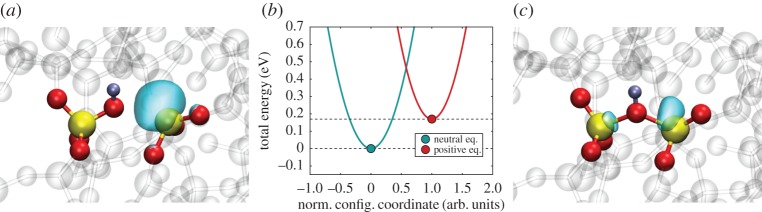
Two charge states of the hydroxyl-E′ centre calculated using DFT, (*a*) neutral and (*c*) positive. H atoms are shown as silver, Si atoms as yellow and O atoms as red. The localized highest occupied orbital is shown as the turquoise bubble for the neutral charge state, whereas it represents the lowest unoccupied orbital for the positive charge state. Note that all atomic positions around the defect change when the charge state is changed. In panel (*b*), the diabatic potentials of the two states are shown qualitatively as a function of the reaction coordinate. In the classical limit of the NMP transition, charge capture takes place at the intersection of the two parabolas. The intersection point determines the barrier that has to be overcome for this reaction.

Conventional RTN and NBTI models assume that a defect can exist in two states: charged and neutral. For instance, in an RTN experiment, the drain current would switch between two discrete current levels, with the transition times being exponentially distributed, consistent with a two-state Markov process. An example of a two-state defect in a-SiO_2_, which is neutral in state 1 and positively charged in state 2 is shown in figure 3. The configuration in figure 3*a* demonstrates the positions of the atoms constituting a neutral hydroxyl-E′ centre (see detailed description below) calculated using DFT and the wave function of the highest occupied state. This defect can trap a hole, which is accompanied by reformation of the second Si–O bond and by a decrease in the separation between the two neighbouring Si atoms, as shown in figure 3*c*. A configuration coordinate (CC) diagram for this defect in figure 3*b* shows schematic harmonic diabatic potentials for the neutral and positively charged states.

However, TDDS studies of 35 defects in six pMOSFETs (*W*×*L*=150×100 nm, 2.2 nm SiON [58]) have demonstrated that many trapping events cannot be explained with a simple two-state model. For example, the emission times are found to be either bias-dependent or bias-independent, a behaviour that also can change when the applied drain bias is changed. In addition, capture and emission times show a much smaller correlation than would be explicable by a two-state model. Finally, charge capture was found to be frequency-dependent, a behaviour that is impossible to explain by a simple two-state model. It has been found [59] that the additional states introduced to explain the first two observations automatically also explain this frequency dependence. In essence, oxide defects can be best described when one assumes that, in addition to a stable equilibrium state, defects also have a metastable state and that thermally activated but field-insensitive transitions are possible between both states [7,11].

**Figure 4.**
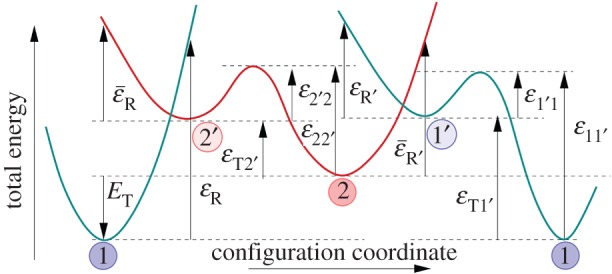
Four-state defect model used to analyse the results of TDDS measurements show a schematic presentation of a cross section of the potential energy surface along a configuration coordinate (CC). The schematic illustrates the energy parameters needed for calculating the rates of vibronic transitions described using NMP theory ($1↔2^{\prime }$ as $2↔1^{\prime }$) and thermally activated transitions described using transition-state theory ($2^{\prime }↔2$ and $1^{\prime }↔1$).

A bistable defect model features quite complicated charge-trapping dynamics, including two-step capture and emission processes. In particular, this means that in addition to the two states shown in figure 3, one has to assume the existence of two metastable states, 1′ and 2′, as shown in figure 4. Two of the states are electrically neutral (1 and 1′), whereas two other states (2 and 2′) are singly positively charged after hole trapping. In each charge state, the defect is represented by a double well, with the energetically lower of the two states being the equilibrium state and the other the closest metastable minimum. Transitions involving charge exchange with the substrate are assumed to occur between 1 and 2′ as well as 2 and 1′. On the other hand, transitions between 1 and 1′ as well as between 2′ and 2 are assumed to be thermally activated transitions between two defect configurations in the same charge state. As mentioned before, such a four-state defect model also allows for different transition paths, which can, for instance, explain the bias-dependent/independent emission behaviour observed in TDDS [11]. Overall, this four-state NMP model (figure 4) has been used successfully to fit a wide range of experimental data [7,26,60,61].

Even though this model was initially developed around previously suggested ideas regarding oxygen vacancies [62–65], it is agnostic to a particular defect and just requires that two states are accessible to the defect initially and after the charge-trapping event. Describing the kinetics of charge trapping/emission by a defect in this model requires 11 parameters to describe the potential energy surface. These correspond to the barriers and relaxation energies of the different processes shown in figure 4. In addition to these parameters, the position of the trap in the oxide, the capture cross section and an attempt frequency should be specified to fully define the transitions [11,56]. These parameters are described in more detail later in the paper. Most of the energetic parameters could, in principle, be obtained from DFT calculations, provided the atomistic structure of the defect is known. Conversely, this procedure allows one to link the defect parameters calculated using DFT to experimental measurements and thus can help to identify the atomistic structure of experimentally visible defects.

**Figure 5.**
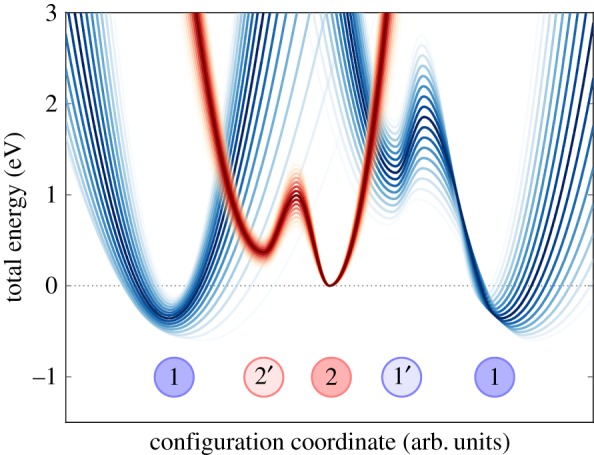
Diagram illustrates the distribution of the potential energy surface parameters shown in figure 4 arising in TDDS experiments. It shows an average CC diagram extracted experimentally for 35 defects using TDDS. Additionally, the envelope curves of the potential energy surfaces for calculated standard deviations of the characteristic parameters are schematically shown up to a deviation of 1.5*σ*.

The largest complication for such an identification is related to the large variations in the local environment of defects in amorphous SiO_2_, as the experimentally measured parameters in CC diagrams (figure 5) contain large variations. One can clearly see that qualitatively similar models give rise to a large variety of possible combinations of relaxation energies and energy barriers. Any comparison of model parameters with experiment therefore must involve statistical analysis. However, from figure 5, one cannot directly deduce the distributions of the particular model parameters. For example, the height of the curves at the barrier $1^{\prime }\to 1$ does not only depend on *σ*_1′1_ but also on *σ*_*E*_T__ and *σ*_*ϵ*_T1′__ of the states 1′ and 1. An alternative representation showing these *σ* can be found in the work of Grasser *et al.* ([58]; figure 4).

In the following, we present the results of DFT calculations of defects suggested as potential candidates responsible for NBTI in Si-based MOSFETs. We first introduce the defect models and discuss the distributions of defect parameters originating from disorder in a-SiO_2_ structures. We then move on to discuss which of these defect models could best explain the volatility in charge-trapping behaviour observed in TDDS measurements.

## 3. Atomistic modelling of hole-trapping defects

Early DFT calculations of defects responsible for reliability issues in Si/SiO_2_ devices focused on crystalline SiO_2_ (α-quartz) structures. In particular, Blöchl & Stathis [66] modelled a number of point defects in α-quartz to assess their role in leakage current. These defects included interstitial hydrogen, oxygen vacancies and their complexes. He concluded that a so-called hydrogen bridge (HB) defect could be involved in electron-transfer processes responsible for leakage current in Si devices containing a-SiO_2_.

Several groups carried out DFT calculations of oxygen vacancies and hydrogen-related defects in a-SiO_2_ [58,64,65,67–69] in the context of RTN and NBTI in MOSFETs. In particular, Schanovsky *et al.* [61] investigated the atomistic nature of the defects that could be involved in NBTI. Using DFT calculations, they examined the oxygen vacancy (OV) and HB defects in periodic cells of α-quartz. The total energies calculated for these defects were then used as parameters for calculating the capture and emission time constants from a Si substrate into the defects and compared with experimental results for NBTI. They concluded that the OV in α-quartz could not be responsible for NBTI as its thermodynamic energy level is too deep to explain the experimental observations.

In the following, we briefly recall the results of *ab initio* calculations of the three defects in *amorphous* SiO_2_ often considered to be responsible for degradation of MOSFETs: the OV, HB and hydroxyl-E′ centre, and then examine whether they can explain the TDDS data. While samples measured in TDDS have a SiON dielectric, the calculations were carried out for the simpler case of a-SiO_2_, in order to minimize the number of possible defect configurations. This choice is justified by the fact that charge capture and emission behaviour in pMOS devices is very similar, regardless of whether the gate oxide is SiO_2_, SiON or a high-k gate stack. This suggests that the defects involved are common to all these materials. One common characteristic of all those oxides is a certain concentration of hydrogen in the material. Hydrogen is abundant in processing of Si-based MOSFETs and is thought to both passivate and modify relevant defects [9,21,70–73]. The calculated properties of the HB and hydroxyl-E′ centre have been compared with the statistically distributed defect parameters obtained from TDDS and it has been concluded that they are likely candidates for explaining the TDDS data [58]. Both defects have the four states required for explaining the trapping behaviour of both RTN and NBTI. Following this brief detour, we investigate whether the mobility of hydrogen in these defects can also explain the experimentally observed defect volatility.

### (a) Details of calculation

The calculations described below make use of both classical force-field and *ab initio* calculations to generate a-SiO_2_ structures. The procedure used to create these structures is described in detail in the work of El-Sayed *et al.* [74]. Here, we briefly describe how these models were created.

The ReaxFF force-field [75] implemented in the LAMMPS code [76] was used to generate 116 periodic models of amorphous SiO_2_, each containing 216 atoms. Starting from β-cristobalite, molecular dynamics simulations were run in order to melt and quench the models. The temperature was raised to 7000 K to melt SiO_2_ within the ReaxFF force-field, followed by a quench to 0 K at a rate of 6 K s^−1^. This procedure was used to create 116 defect-free continuum random network a-SiO_2_ structures. A barostat was used to keep the pressure fixed at 0 bar. Densities of the ReaxFF a-SiO_2_ structures ranged from 1.99 to 2.27 g cm^−3^, averaging at 2.16 g cm^−3^. These values fall within the range of densities known for a-SiO_2_.

DFT, as implemented in the CP2K code [77], was used to further optimize the ReaxFF structures and calculate their electronic structures. In order to minimize the errors in the energy levels and band gaps, the non-local, hybrid functional PBE0_TC_LRC, which contains 20% Hartree–Fock exchange was used in all calculations. A cut-off radius of 2.0 Å was used for the truncated Coulomb operator [78]. A double-*ζ* Gaussian basis set with polarization functions [79] was employed in conjunction with the Goedecker–Teter–Hutter (GTH) pseudo-potential [80]. To reduce the computational cost of non-local functional calculations, the auxiliary density matrix method (ADMM) was employed [81]. All geometry optimizations were performed using the Broyden–Fletcher–Goldfarb–Shanno (BFGS) [82–85] algorithm to minimize forces on atoms to within 37 pN (2.3×10^−2^ eV Å ^−1^). Cell vectors were not allowed to relax from their ReaxFF values. The calculated structural parameters of amorphous structures including the average structure factor have been discussed in previous work [86]. The structure factor peaks of both the ReaxFF and DFT optimized models agree very well with the experimental data, indicating that the medium- and long-range order of the models is well described by these models.

After the geometry optimization, all 116 defect-free ReaxFF structures were used to study the interaction of H with the a-SiO_2_ network. Oxygen atoms were removed from a single a-SiO_2_ structure one by one to create 144 neutral oxygen vacancies. Energy barriers between different defect configurations were calculated using the climbing-image nudged elastic band method (CI-NEB) [87,88]. Linear interpolation was used to generate 10 images between an initial and final configuration to be used as the band in the CI-NEB trajectory for each calculated barrier, with each of the images connected by a spring with a force constant of 2 eV Å ^2^.

In the following, we briefly describe the structures of the three defects and discuss their properties.

### (b) Oxygen vacancy

**Figure 6.**
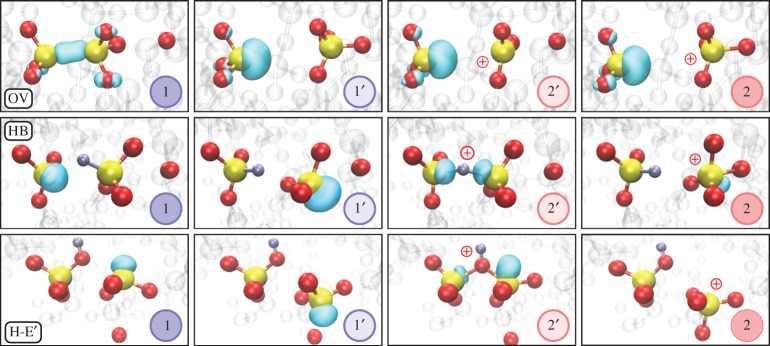
Atomic configurations corresponding to states 1, 1′, 2′ and 2 for the oxygen vacancy (OV, top row panels) hydrogen bridge (HB, middle row panels) and the hydroxyl-E′ centre (H-E′, bottom). H atoms are shown as silver, Si atoms, yellow and O atoms, red. The turquoise bubble represent the localized highest occupied orbitals for the neutral charge states and the lowest unoccupied orbital for the positive charge states. Upon hole capture, the defect can go into state 2′ and the Si atoms move closer together in all the defects. Depending on the gate bias, the defect either goes back to state 1 or, eventually into the positive state 2 or the neutral state 1′, where the right Si has moved through the plane of its three O neighbours, forming a puckered configuration by bonding to a neighbouring O in the right.

We begin our discussion with the most commonly studied defect in silica, the OV. An OV forms when a two-coordinated oxygen atom in the SiO_2_ network is missing. It is often assumed that this diamagnetic defect gives rise to an optical absorption band with a maximum at around 7.6 eV relative to the valance band in both crystalline quartz and in a-SiO_2_. In the most stable configuration, the two Si atoms neighbouring the vacancy displace towards each other and form a bond accompanied by a very strong relaxation of the surrounding silica network (figure 6 OV 1). Upon trapping a hole, the OV converts into a paramagnetic E′ centre, which is the most abundant dangling bond centre in a-SiO_2_ [89] and has been investigated in a number of papers [64,65,68,73,90–102]. In a-SiO_2_, this defect has several configurations, dependent on the local environment [64,65,68,73,93,94,99–102]. One of the best studied metastable configurations is formed when the Si ion with the hole moves away from the other silicon atom through the plane of the three neighbouring O atoms and is stabilized by the interaction with another, so-called back-oxygen ion [65,73,93,99] (figure 6 OV 2). It has been attributed to the defect known as the E${\;}_{\gamma }^{\prime }$ centre and is also referred to as the *puckered* configuration. It is also metastable in the neutral state of the OV (shown in figure 6 OV 1′) giving the four required states for this defect to be relevant for the defect model described above. However, the back-oxygen ion is not always in the right position in a-SiO_2_ to stabilize the puckered configuration 2 (see discussion in [64,99]) and so this configuration does not exist at every Si site in a-SiO_2_. In our set of 116 a-SiO_2_ structures, a stable four-state configuration shown in figure 6 was found in ≈6% of the possible defect sites, this is a density of possible four-state defect sites of 2.6×10^21^ cm^−3^. Note that these are the maximum *possible* sites without taking the defect’s energetics into account. The actual defect concentration will be dictated by its energetics.

**Figure 7.**
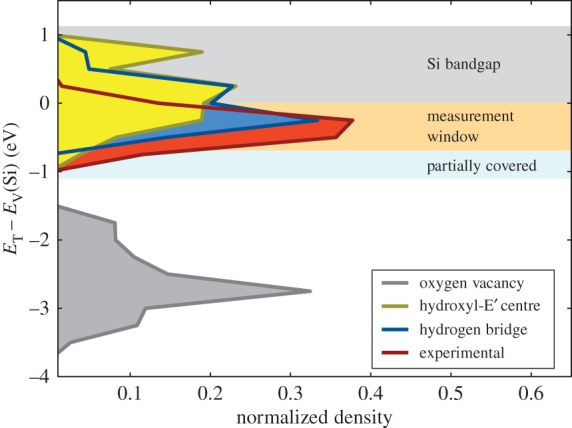
Distribution of the thermodynamic charge-trapping levels, *E*_T_, a fundamental parameter that decides on which trap can be charged for a combination of certain stress- and recovery voltages. The top of the Si valence band is set to zero. Note that all defects close to the valence band of Si, *E*_V_(Si), will contribute to RTN in a pMOS. Clearly, the OV/E′ centre is too low in energy, whereas both the hydrogen bridge and the hydroxyl-E′ centre are in good agreement with the data inside the experimental window. Recall the uncertainty in DFT energy-levels and the −0.4 eV energy shift used.

Despite the OV having the four required states for our model, it has been shown by Schanovsky *et al.* [61] and Grasser *et al.* [58] (also see figure 7) that the trap level (*E*_v_(Si)−*E*_T_≈−3.5 eV) for this defect lies too low to be charged during typical measurement and operating conditions. As shown below, the hydrogen-related defects better satisfy these requirements.

### (c) Hydrogen bridge defect

The HB forms when a two-coordinated oxygen atom in the SiO_2_ network is replaced by a hydrogen atom (or, equivalently, when a hydrogen atom is trapped in a pre-existing neutral OV). In order to study the interactions of hydrogen with vacancies in a-SiO_2_, all oxygen atoms in a single a-SiO_2_ structure were removed one by one to create 144 configurations of the OV. An H atom was then placed next to each vacancy and the geometry optimization resulted in an asymmetric defect structure in which the H is closer to one of the vacancy’s Si atoms (figure 6 HB 1). This is manifested as a short Si–H bond and a longer range Si⋅⋅H interaction, where ⋅⋅ indicates a non-bonding interaction. The short Si–H bond averages at 1.47 Å, ranging from 1.44 to 1.51 Å. The distance of the Si⋅⋅H interaction averages at 2.21 Å and ranges from 1.74 to 3.13 Å, indicating that the shorter bond is a strong chemical bond while the longer range Si⋅⋅H interaction is weak and strongly influenced by the amorphous environment (see reference [103] for further discussion). The Si–O bonds associated with both of these Si atoms average at 1.63 Å and have a range of just under 0.04 Å. These Si–O bonds are only slightly longer than other Si–O bonds in the system which indicates that the relaxation is localized at the defect centre. The unpaired electron of the vacancy is localized on the Si atom not possessing the hydrogen (figure 6)

The HB also has a puckered configuration in its positively charged state 2 and a secondary configuration in the neutral state 1′ (figure 6 HB). Unlike the puckered configuration of the OV, in this case, the Si atom with the unpaired electron is inverted through the plane of the three neighbouring oxygen ions with a dangling bond facing towards a back-oxygen ion again (a so-called back-projected configuration of the Si dangling bond). All four configurations of the HB are, however, stable in 55% of the investigated structures, this is a density of possible four-state defect sites of 2.3×10^22^ cm^−3^. Note that these are again the maximum *possible* sites, as discussed above for the OV.

### (d) Hydroxyl-E′ centre

It has recently been demonstrated that the hydroxyl-E′ centre (H–E′ centre) forms when an H atom interacts with strained (>1.65 Å) Si–O bonds in a-SiO_2_ network [74,103] (this criterion is fulfilled for ≈2% of the Si atoms in our structures [103]). Such bonds do not exist in α-quartz, and this defect only forms at particular sites in amorphous structures. Briefly, it resembles an E′ centre, i.e. a three-coordinated Si atom with an unpaired electron [93], facing a hydroxyl group (figure 6 H-E′ 1). The Si dangling bond introduces a one-electron state located on average 3.1 eV above the a-SiO_2_ valence band, ranging from 2.40 to 3.90 eV, making the defect level almost resonant with the top of the Si valence band in some configurations. The hydroxyl-E′ centre also has a stable puckered configuration in the positive state 2 and a stable back-projected configuration in the neutral charge state 1′ (figure 6 H-E′).

Owing to the favourable position of the hydroxyl-E′ centre’s energy level, it can trap a hole under typical MOS operating conditions. We calculated the hole-trapping configurations in 61 a-SiO_2_ structures and found that two types of stable configurations exist [104] for ≈7% of the strained (greater than 1.65 Å) Si–O bonds, this gives a density of *possible* four-state defect sites of 2.8×10^19^ cm^−3^. The first configuration reforms a weak Si–O bond at the three-coordinated Si, forming a hydronium-like structure. This can be seen in configuration 2′ in figure 6 H-E′. The hole is localized around the bridging O. A back-projected configuration is formed when the three-coordinated Si moves through the plane of its O neighbours and forms a weak bond with a two-coordinated O. The hole in this configuration is highly localized on the inverted Si. This configuration is shown as configuration 2 in figure 6 H-E′. Thus, the hydroxyl-E′ centre also exhibits the bistability required for the four-state NMP model.

## 4. Statistical analysis

The structural disorder in a-SiO_2_ results in wide distributions of defect parameters in experimental measurements as well as in DFT calculations. Linking the experimental and theoretical data therefore requires comparing their statistical properties. Using the four-state model described in §2b, we extract the corresponding parameters from TDDS measurements and compare them with the parameters obtained in DFT calculations for defect candidates in a-SiO_2_. If the distributions of parameters match those for a particular defect, we consider this defect to be a likely candidate for the experimentally observed charge capture and emission effects.

We calculated a number of different defect properties in a-SiO_2_. These include the thermodynamic defect levels, energy barriers and defect relaxation energies required by the four-state NMP model. For some of these properties, we have obtained large datasets and where appropriate we use statistical descriptors to provide an idea of the shapes of the distributions we obtain. However, some datasets are rather limited, such as the calculated barriers, and we do not attempt to provide a complete statistical view of these data. We note that owing to computational limitations these statistics are not meant to be representative of the entire population of each defect in a-SiO_2_, but rather offer a statistical view of the defects that we have studied using the methods described above.

One of the most important parameters of any defect is the distribution of thermodynamic energy levels, *E*_T_, shown in figure 7. In our experimental data, only defects roughly between −1.0 and 0.0 eV below the valence band maximum of Si, *E*_v_(Si), are accessible under typical experimental conditions. Unfortunately, DFT energy levels contain some uncertainty, making accurate assignment of levels difficult. In our case, one can relate the defect levels to *E*_v_(Si) calculated using the same hybrid functional. This would place 60%/75% of our HBs/hydroxyl-E′ centres above *E*_v_(Si) and thus render them permanently positive under NBTI conditions. To retain a larger fraction of our defect population (58%/50%) and improve our statistics, we introduced an energy correction of −0.4 eV, corresponding to ≈50% of our SiO_2_ band gap error (0.8 eV). Applying this adjustment, the HB and the hydroxyl-E′ centre are in the right energetic position below *E*_v_(Si). In the crystalline SiO_2_ structures, the trap level for the OV has already been shown to be much too deep for OV to be a viable candidate [58,61]. Using an amorphous host material for the calculation does not change this observation. The corresponding distribution of the trap-levels can be seen in figure 7.

**Figure 8.**
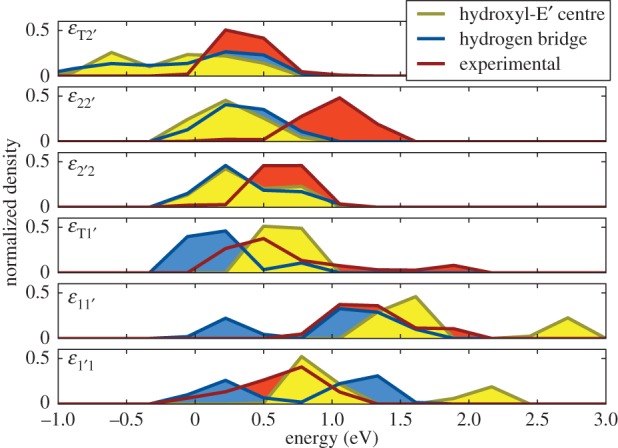
The experimental and calculated barriers for the various transitions in the four-state model. The parameters are shown on the diabatic potential energy diagram in figure 4. While overall good agreement is obtained, the theoretical barrier *ε*_22′_ is too small in general. Note that the defects with negative *ε*_T2′_ are two-state defects at the border of our experimental window.

The experimental data suggest that the defect distributions are much wider than what can be captured in our TDDS window [105,106]. Because in our relatively small sample, no theoretical defect could be expected to exactly fit a particular experimental defect, we always have to compare their distributions. The distributions of the HB and the hydroxyl-E′ are generally in reasonable agreement with the energy barrier distributions extracted from the experimental data (figure 8). The most significant deviation is observed for the barrier between states 2 and 2′, *ε*_22′_. This barrier determines the hole emission time constant, but the calculated distribution is notably smaller than the experimental values, on average by 0.5 eV. Whether this is an artefact of our bulk amorphous oxide structure or evidence for a different microscopic nature of the defect remains to be clarified.

We therefore conclude from the statistical comparison that the OV is a very unlikely candidate for explaining charge capture and emission in our TDDS experiments. The statistical properties of the HB and the hydroxyl-E′ centre, on the other hand, give a good match for the majority of the parameters (figure 4) of the four-state model. Because the statistical properties are very similar for both, it is not possible to deduce from these data whether one of them is more likely than the other. It cannot be ruled out that both defects could contribute to the experimentally observed charge capture and emission events. However, the experimental observations in §5 provide further clues supporting the hydroxyl-E′ centre.

## 5. Defect volatility

**Figure 9.**
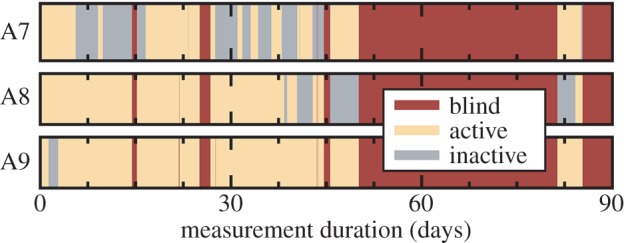
TDDS measurements for three selected defects which were monitored over three months. The plots show when the defect is electrically active or inactive (volatile). Occasionally, the experimental conditions did not allow for an observation (‘blind’ phases), for instance during a long high-temperature bake around the beginning of the third month.

As shown earlier, RTN and TDDS analyses have provided a deep insight into the trapping dynamics of oxide defects. However, one additional feature that is observed during these measurements has not yet been addressed in detail. As mentioned in Introduction, defects have been found to frequently dis- and reappear in the measurements (figure 9), and can sometimes even disappear completely from our observation window [21,107]. This so-called *volatility* is not a rare event, but can potentially occur for a majority of the defects, particularly when electrons are injected into the oxide. A consistent model of oxide defects must therefore not only describe their behaviour when electrically active, but also allow for them to dis- and reappear during measurement cycles.

In our TDDS measurements, we observe time constants for defect signals disappearing in the volatile state, *τ*_v_, typically in the range of hours to weeks. The upper limit is clearly limited by the measurement time. The lower limit has not yet been rigorously tested. We speculate that a *τ*_v_ as low as 1 s could well be detected for a defect normally capturing and emitting in the microsecond regime. However, up to now, the lowest observed *τ*_v_ has been 20 min. Assuming that the dynamics is determined by a thermally activated rearrangement of the atomic structure, we are again dealing with a two-state process (active/inactive). Similar to RTN and 1/f noise, we can estimate the corresponding reaction barrier with an Arrhenius law [1,11,41]

5.1 $$\frac{1}{\tau_{v}}=\nu \cdot e^{E_{B}/k_{B}T}.$$

Assuming an attempt frequency of *ν*=10^13^ s^−1^ [7,58], the corresponding rearrangement barrier height *E*_B_ at room temperature should be about 1.0 eV. This value only increases to about 1.2 eV for a *τ*_v_ of one month. Of course, higher barriers can be overcome when measuring at higher temperatures and for longer times.

As was shown in §3, both the HB and hydroxyl-E′ centres exhibit the bistability and the trap level positions favourable for RTN and NBTI observed in Si MOSFETs. Because both of them contain a hydrogen atom and several publications have shown that hydrogen can be released during electrical stress [71,108–112], we investigated whether the dynamics of the hydrogen atom could be a possible cause of volatility. For example, the presence of the hydrogen atom in the HB moves its level into a more favourable position for hole trapping with respect to the OV. Thus, losing hydrogen could take the defect out of the TDDS measurement window. This H relocation corresponds to either a neutral hydrogen atom moving away from the neutral defect state or a proton from the positive defect state. Therefore, in the following. we consider the two hydrogen-containing defects in both charge states.

### (a) Hydrogen bridge

To describe the disappearance of the hydrogen-containing defects, the hydrogen has to move away from the defect to form a configuration that we refer to as precursor configuration 0. We start by considering the release of a neutral hydrogen atom from the HB into an interstitial position. This reaction can be viewed as a two-step process. First, the Si–H bond should be broken, followed by the formation of an OV. However, calculating a barrier for the first step is difficult as hydrogen in any interstitial position in the vicinity of the vacancy spontaneously returns into the HB configuration, making it very hard to use the NEB method. If, however, there is a strained bond nearby, the hydrogen binds to a bridging oxygen, then forming a new neutral hydroxyl-E′ centre. The resulting configuration is lower in energy than the interstitial [74] and can therefore be used to calculate the lower limit for the energy barrier for hydrogen atom dissociation from the HB centre. Our calculations show that this barrier is on average 2.6 eV with *σ*=0.67 eV, which is still too high to explain the majority of the data. We therefore conclude that the characteristics of the HB in its neutral charge state are unlikely to change as a result of hydrogen dynamics in such a way as to explain defect volatility.

**Figure 10.**
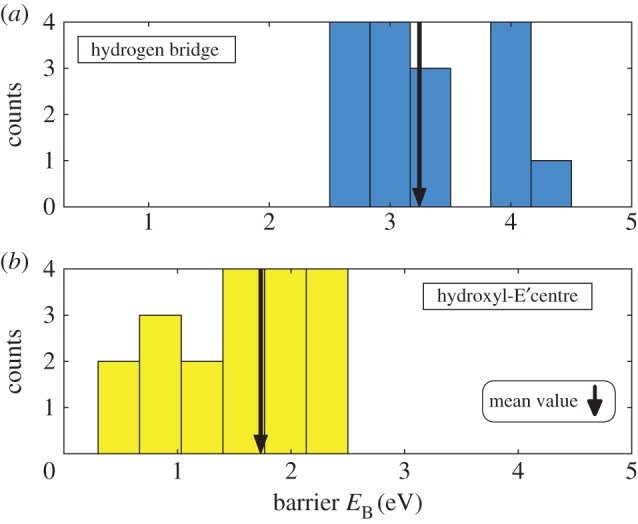
Barriers *E*_B_ from NEB-calculations for the transition $2^{\prime }\to 0^{+}$ (in the case of the HB also $2\to 0^{+}$) into the electrically inactive state 0^+^. For the HB (*a*) even the lowest values found are much too high to be able to explain the observed volatility. Even though the mean value for the barrier height (black arrow) for the hydroxyl-E′ (*b*) is very high too, one can also find very low barriers that could easily be overcome during experimental conditions, giving a possible explanation for defects becoming volatile.

From the positively charged HB, a proton can be released by displacing away from the vacancy to bind to the next available neighbouring oxygen, similar to the proton hopping described in the work of Karna *et al.* [89] and Blöchl [73]. This proton movement to the neighbouring oxygen atom yields a new configuration which we call 0^+^ in its positively charged state. To explore possible sites for proton binding, the proton was moved from its defect position close to a neighbouring oxygen atom (to a distance of 0.8 Å), and the geometry of the system was optimized. We then used the NEB method to calculate the barriers for this proton relocation. For the positively charged case, our NEB calculations on selected transitions for the HB yield reaction barriers for $2^{\prime }\to 0^{+}$ with a minimum of 2.54 eV (or $2\to 0^{+}$ with a minimum of 3.03 eV, respectively). These values are much too high to explain the volatility of the defects (see figure 10 top for the barrier value distribution). Thus, in both charge states of the HB, the calculated barriers are too high to explain the volatility seen in experiments.

### (b) Hydroxyl-E′ centre

In previous work [74], we have shown that the mean barrier for dissociation of the neutral hydroxyl-E′ centre and formation of an interstitial H atom is 1.66 eV with *σ*=0.37 eV. This is already much lower than for the HB, but still slightly too high for this reaction to satisfactorily explain volatility.

#### (i) Proton relocation to a neighbouring oxygen atom

**Figure 11.**
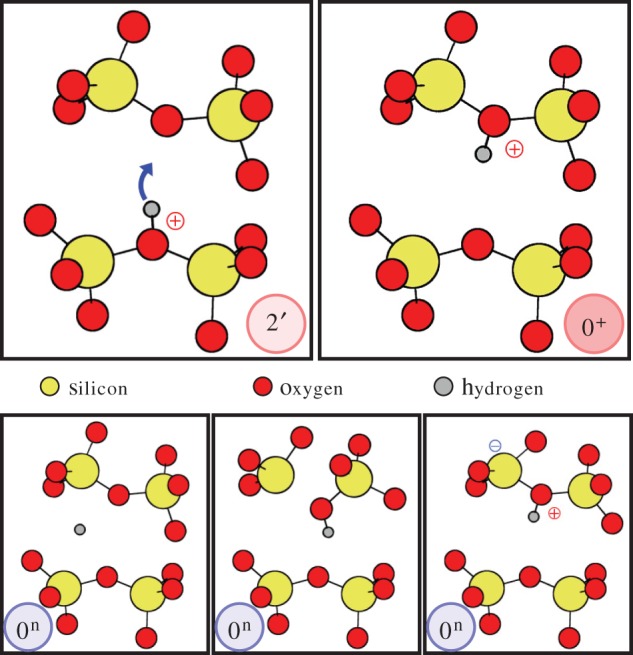
Top: schematic shows the relocation of the proton for the case of the hydroxyl-E′ centre. The proton moves from the defect site in state 2′ (left) onto a neighbouring bridging oxygen atom (right). Owing to the amorphous nature of the structure, in general, the new location does not favour the creation of a new defect. We name this new positively charged state 0^+^. Bottom: when the state 0^+^ is charged neutrally, three different possible states 0^n^ have been found: the H atom becomes interstitial (left), the H atom causes one of the oxygen-silicon bonds to break (middle), forming a new hydroxyl-E′ centre or the H-atom remains attached (right). The latter is only possible when the hydrogen can transfer its electron to an electron-accepting site nearby.

The second possible reaction would again be the relocation of the proton in the positively charged configurations 2 and 2′ onto a neighbouring bridging oxygen atom. For this reaction calculations, Wimmer *et al*. [113] demonstrated that the reaction barriers involving positively charged states (figure 11 top) are considerably lower than for the neutral case. We will therefore focus on this proton relocation.

The NEB calculations performed for the positively charged hydroxyl-E′ centre clearly showed that the reaction $2^{\prime }\to 0^{+}$ is always preferred over $2\to 0^{+}$[113]. Even though the mean value of these transition barrier still lies quite high (1.73 eV), much lower barriers, in the range of 1.0 eV and smaller, were found for the transition $2^{\prime }\to 0^{+}$ for the hydroxyl-E′ (figure 10 bottom). We should note that the states 2′ and 0^+^ of the hydroxyl-E′ are nearly isoenergetic, with 20% even being lower in state 0^+^ [113]. This means that the reverse reaction barriers (and time constants) $0^{+}\to 2^{\prime }$ back to defect activity would be in the same range as the forward reaction $2^{\prime }\to 0^{+}$. This is in agreement with the TDDS measurements [18], making the hydroxyl-E′ centre a plausible candidate for explaining volatility effects.

**Figure 12.**
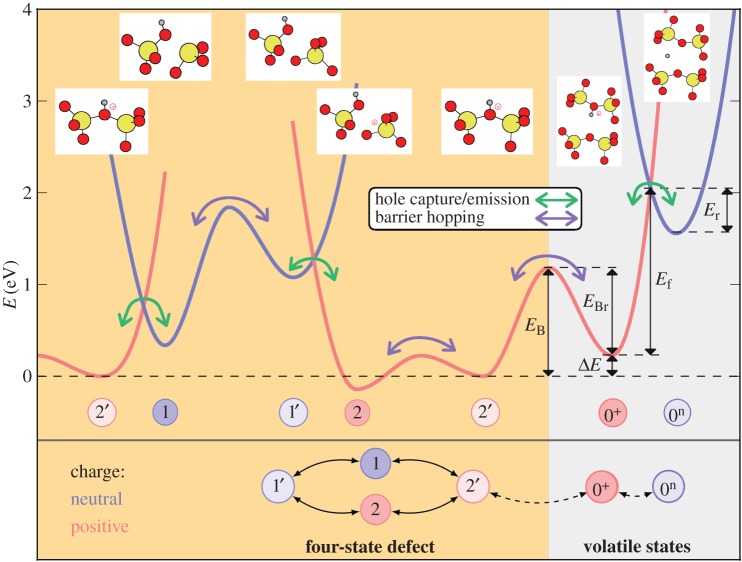
Example of a potential energy surface of a hydroxyl-E′ centre defect along the reaction coordinates between different states. Possible transitions can occur by charge capture or emission (green arrows) or barrier hopping (purple arrows). The defect is electrically active when on the left side of the plot (orange). When it overcomes the barrier $2^{\prime }\to 0^{+},$ it is electrically inactive (grey) and therefore, in general, not visible in the measurements (given certain conditions for the barrier between the states 0^+^ and 0^n^ as described in the text). Depending on the applied gate bias, the parabolas of the neutral states (blue) will be shifted up or down along the energy axis, thereby changing the barriers and time constants for charge-trapping and emission. Note that in this extended model there are now three possibilities to leave state 2′ (to 1, 2 or 0^+^).

An extended model of possible transitions between different defect states is schematically depicted in figure 12. Based on the results discussed above, we assume that the transition into the inactive state is $2^{\prime }\to 0^{+}$. Figure 12 is an extension of figure 4 including volatility. Similar to figure 4, the transitions involving charge transfer are considered in the NMP model. The transitions between states of the same charge are again assumed to be purely thermally activated as well as the volatility transition $2^{\prime }\to 0^{+}$. As described in §2b, the applied voltage moves the neutral (blue) and positive (red) parabolas relative to each other, thereby changing the barriers for the NMP transitions.

However, it has to be clarified whether the defects really would be invisible in our measurements when in the volatile state. For example, if the defect was stuck in state 0^+^, then it would clearly be electrically inactive. This depends not only on the transition barriers back to 2′, which we just discussed above, but also on the emission barrier to the neutral state 0^n^. We will now discuss the effect of barriers for these processes on the results of RTN and TDDS measurements.

### (c) How defects in volatile states behave in RTN and TDDS measurements?

Having ruled out the HB as a possible candidate for explaining volatility, we will focus only on the hydroxyl-E′ centre. It has three different possibilities to relax into the neutral state 0, referred to as 0^n^ (figure 11 bottom). The hydrogen atom can either become interstitial, break one of the bonds at the bridging oxygen (resembling state 1 in figure 6), or just stay attached. The first case is of great importance, because it could also provide an explanation for defects disappearing completely during the measurements if the interstitial hydrogen diffuses away. The last case, where the hydrogen stays attached to the bridging oxygen is only possible if the hydrogen can donate its electron to an electron-accepting site nearby. This could be, for example, a silicon with wide O–Si–O bond angle [86].

Up to now, we have only discussed the transitions to the positively charged state 0^+^. However, reaction barriers between the states 0^+^ and its neutral variant 0^n^ are also of great interest for determining whether the defect would be electrically active in state 0. The dynamics of the defect are determined by the barriers between the states 2′ and 0^+^ (*E*_B_ and *E*_Br_) and by the barriers between the states 0^+^ and 0^n^ (*E*_f_ and *E*_r_). *E*_B_ and *E*_Br_ were calculated using the CI-NEB method, as described in the previous section. *E*_f_ and *E*_r_ can be determined in the classical limit of NMP theory (see §2b) by the intersection point of their potential energy surfaces (figure 12).

First, let us assume that the barriers *E*_B_ and *E*_Br_ are high compared with the barriers that have to be overcome when cycling between the four active states (1, 2, 1′ and 2′) of the model. This holds true for the majority of the investigated defects, therefore volatility time constants are considerably higher (by several orders of magnitude) than the charge capture and emission time constants of the active defect.

We then have to distinguish between two cases: if the barrier *E*_f_ between the states 0^+^ and 0^n^ is higher than the barrier *E*_Br_ back to the state 2′ (figure 12), or has at least the same height, then this would leave the defect electrically inactive in 0^+^. Any charge capture or emission event in state 0 would occur with a similar or lower frequency as volatility itself.

**Figure 13.**
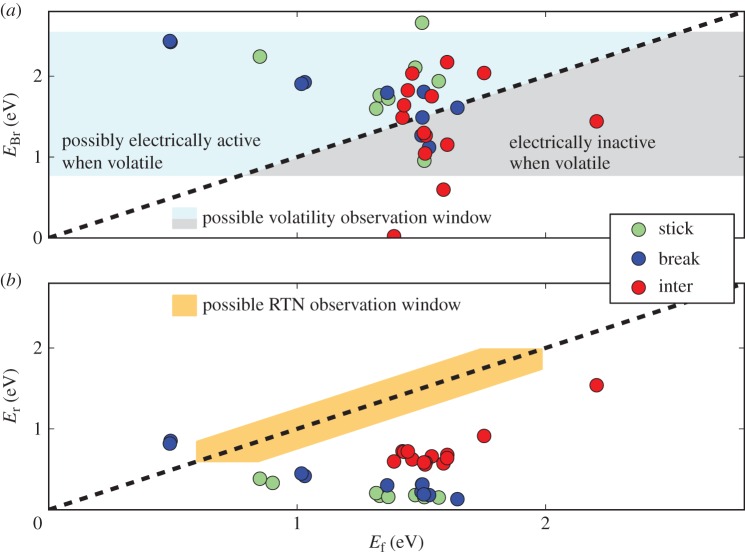
Correlations of the barrier heights *E*_Br_ and *E*_r_ with respect to *E*_f_ (figure 12) for the hydroxyl-E′ centre. We distinguish between the three possible states 0^n^ (figure 11 bottom): the hydrogen atom can either stay attached (stick), break one of the bonds at the bridging oxygen (break) or become interstitial (inter). For about two-thirds of the defects *E*_Br_ is higher than *E*_f_ (*a*). Even though this means that defects should be electrically active, this does not mean that they would be visible in RTN measurements. This is due to the height of *E*_f_ and, even more importantly, owing to the large difference between *E*_f_ and *E*_r_ (*b*), which moves them far outside of the RTN detection window. However, as discussed in the text, those defects should be visible in TDDS, but the signal would most probably not be identified as being related to the initial defect.

The second case occurs if the barrier *E*_f_ is indeed lower than *E*_Br_. This is the case for about two-thirds of the DFT defects (blue part in figure 13 top). Such a defect could be electrically active in the volatile state as well. However, for all the calculated defects, the barriers *E*_f_ and *E*_r_ are found to be of very different height (typically *E*_f_ ≫*E*_r_, figure 13 bottom). Therefore, the defect could, indeed, be electrically active, but at the same time hardly observable in RTN measurements owing to its very short time in the energetically higher state [11]. For the observable RTN measurement window in figure 13 bottom, we assumed a maximum ratio of capture and emission times by a defect of 100 and a minimum such ratio of 0.01. The window is further limited by minimum time constants of 1 ms and a maximum of 1 ks.

It must be kept in mind that in TDDS, owing to different emission times, this kind of defect would theoretically be visible, but as a different cluster to the initial defect in the spectral map (figure 2). However, owing to the different emission times this new cluster would show up on the maps with a similar step height but at a different time. Therefore, it would most probably not be associated with the original defect in such a spectral map.

We can therefore conclude that the vast majority of our calculated hydroxyl-E′ centres would be invisible in measurements when in the volatile states, assuming the validity of the model in figure 12. The hydroxyl-E′ centre is therefore a good candidate to explain not only the electrically active branch, but also the volatility effect. Furthermore, as discussed above, we also found that there are defects that eventually could release their hydrogen atom. This could be a possible mechanism to explain the complete disappearance of defects in our measurements. It also provides a possible link to hydrogen release during electrical stress [13,71,108–112].

## 6. Conclusion

Recent technological advances have led to shrinking of MOSFETs down to a size where there are only a small number of oxide defects per device. As a consequence, the device degradation is now determined by the response to single defects. On the other hand, this has opened opportunities for new measurement methods like TDDS that allow one to study individual defects in much greater detail than ever before.

To explain the experimental data for charge capture and emission into oxide defects in MOSFETs, we have proposed a four-state model and extended it by two additional states to explain the volatility observed in our measurements. We have shown that the model is able to explain the complicated defect dynamics. Based on this model we then investigated three defect candidates: the OV, the HB and the hydroxyl-E′ centre. Owing to the amorphous nature of the oxide, the properties of these defects are statistically distributed. A comparison can therefore only be made on a statistical basis. The data obtained from density functional theory calculations in a-SiO_2_ were compared with the statistical properties deduced from experiments. We concluded that the OV is a very unlikely candidate because its thermodynamic trap level lies too low to be charged during typical measurement conditions. However, the HB and the hydroxyl-E′ centre provided a much better agreement with the measurements.

Finally, we investigated whether the experimentally observed volatility could be satisfactorily explained by the dynamics of hydrogen atoms embedded in these two defects. We showed that hydrogen relocation onto a neighbouring oxygen atom is a possible mechanism to explain this effect. However, this only holds true for the hydroxyl-E′ centre. For the HB, all calculated reaction barriers for the possible volatility-related reactions appear too high for a satisfactory explanation.

We can therefore conclude that, based on the suggested model, the OV is a very unlikely candidate to explain the measurement data. The hydroxyl-E′ centre, on the other hand, is a promising candidate to explain all the observed features including volatility. The HB could still explain experimental data not showing volatility. Our results highlight a complex interplay of electron capture/emission and thermally activated hydrogen motion in oxide films.

## Data accessibility

Simulation data referred to in this work can be accessed at https://owncloud.tuwien.ac.at/index.php/s/ZAil9YjcpB40QOV.
